# D2A: a community-led smartphone tool for malnutrition screening in Kenya

**DOI:** 10.3389/fpubh.2025.1695850

**Published:** 2026-02-27

**Authors:** Ravi Bhavnani, Nina Sophia Link

**Affiliations:** Department of International Relations & Political Science, The Geneva Graduate Institute, Geneva, Switzerland

**Keywords:** child malnutrition, community-led, innovative, screening, smartphone, low-cost

## Abstract

Timely assessments of child acute malnutrition are essential for effective treatment and prevention of undernutrition. We developed a simple smartphone app, *D2A* (“Data to Analysis”), to enable regular self-collection of Family MUAC (Mid-upper Arm Circumference) and key household drivers of wasting by mothers and caregivers. Based on a seven-month pilot study with 180 households, we explore the acceptance, accuracy, and cost of app-based self-collection of Family MUAC with and without the assistance of Community Health Volunteers (CHVs) relative to paper-based nutrition screenings. Results indicate: (i) similar classification accuracy of wasted children; (ii) no difference in participant dropout rates and a 15% higher completion rate for CHV-assisted reporting by households (compared to no assistance); (iii) lower cost for app-based collection amortized over seven months. Preliminary evidence suggests that self-reporting by households via smartphone apps constitutes a feasible alternative to less frequent, more costly paper-based nutrition screening, the latter more susceptible to interruption in remote, hard-to-access areas.

## 1 Introduction

While Kenya is ranked high in its efforts to end hunger and improve nutrition (1), approximately 12% of Kenyan children under five years were acutely malnourished in the first half of 2025 (2). Child acute malnutrition, also referred to as wasting, and defined as the condition of being significantly underweight for one's height (3, 4), is life-threatening and caused by insufficient nutrient intake and absorption (5, 6). Affected children with weak immunity can become dangerously thin in a matter of days, with the risk of mortality heightened in the absence of treatment (7–9).

When acute malnutrition spirals, treatment of the condition becomes increasingly difficult, raising the likelihood of relapse (10). To avert famine, stakeholders need to mitigate immediate harm and respond to humanitarian needs in a timely and targeted manner (11, 12). While longitudinal evidence constitutes the gold-standard for decision-making and intervention, improved forecasts with adequate lead-time are also key (13). Several recent efforts have resulted in the development of sophisticated models (13–18) that employ granular data for preventive action, identifying the fundamental drivers of child malnutrition and anticipating which households are more likely to be at risk. Yet these efforts require reliable, consistent, and near-real time data, in order to move beyond less transparent, more time-consuming, labor-intensive and costly ‘convergence-of-methodology' approaches employed by current early warning systems—FewsNet and the Integrated Phase Classification (IPC) in particular (15, 19).

It follows that in many regions, evidence of acute malnutrition remains fragmented, and where data exists it is often not sufficiently granular to inform decisions (11, 20, 21). Conventional, paper-based nutrition assessments—such as SMART (22) or Demographic Health Surveys (DHS) (23)—are costly and logistically challenging (24, 25), making frequent implementation difficult (26). And with limited resources, regular nutrition screenings rarely extend beyond the duration of a project (27), with still fewer existing initiatives tracking the same children longitudinally (28). Conventional methods also face disruptions in terms of access during public health emergencies, like COVID-19 (29, 30) or periods of armed conflict and violence. In times of crisis, enumerators are often unable to reach remote communities (31–33). Thus, precisely when the need for information is acute and essential for timely identification, thorough treatment, and fine-grained predictions of wasting, paper-based nutrition screenings come to a halt (34).

To fill this gap, we developed *D2A* (“Data to Analysis”), a simple smartphone survey deployed via *KoboToolbox* and designed for community-led child malnutrition screening. Self-collection of Family MUAC and entering information about key household-level correlates used to predict the risk of wasting into *D2A* takes less than five minutes, with no requirement to be able to read or write. *D2A* features audio-guides in three different languages as well as video-instructions, and allows users to take pictures or select simple emojis in response to survey items. As soon as a smartphone connects to the internet, self-reported Family MUAC and key household correlates are automatically uploaded and saved in a secure server. Given that community-led malnutrition screening via smartphone apps remains the exception rather than the rule, we tested *D2A* in a seven-month pilot study with 180 households in West Pokot County, Kenya. Existing partnerships with local authorities, combined with the region's predominantly arid and semi-arid (ASAL) climate and persistently high rates of child acute malnutrition provided a suitable and feasible study environment. We employ mixed methods to: (i) assess the accuracy of *D2A* relative to pen-and-paper monitoring; (ii) evaluate household acceptance of autonomous app-based nutrition reporting across livelihood contexts, with and without support from Community Health Volunteers (CHVs); and (iii) compare the costs of *D2A* with monthly enumerator-based pen-and-paper surveys over a period of 12 months. Qualitative insights from semi-structured expert interviews and focus group discussions (FGD) with study participants complement quantitative group comparisons.

## 2 Mobile nutrition monitoring

Near-real time assessments of child nutrition, especially in times of humanitarian crisis, require innovative, low-cost data collection techniques and we are by no means the first to propose the use of mobile phones in this regard (19, 25, 26, 35–38). Mobile-based food security monitoring systems may be broadly classified by whether they are limited to data collection or more interactive in nature. Data collection usually relies on telephone interviews with either human or computer operators (39) and to a lesser extent on unstructured supplementary service data (USSD) increasingly used to analyze movement and mobility patterns (40). Interactive surveys, like *mNutrition* (41) or the Mobile Vulnerability Analysis and Mapping (*mVAM*) project (36), collect information while providing feedback and recommendations to households, typically via phone calls and text messages or by means of short video clips (42). Yet, weight, height, Mid-Upper Arm Circumference (MUAC) and other anthropometric measures required to diagnose child acute malnutrition are not considered in any of the above mobile-based food security systems. The use of smartphone-based acute malnutrition screenings, by contrast, is relatively isolated. We are aware of one study that tested smartphone surveys with healthcare staff to develop a child nutrition surveillance system in the Republic of Mauritius (43), and of another project that recently explored autonomous Family MUAC screenings via smartphone apps in rural Kenya (19). Jensen et al. (19) demonstrated the potential of household-led Family MUAC screening and reporting via smartphone apps for fast data synchronization and near real-time information transfer, as well as the possibility of tracking child acute malnutrition at high-frequency and over an expanded period of time. To date, no initiative (including the two mentioned above) has resulted in the self-collection of Family MUAC alongside a minimally requisite set of household correlates and other drivers of child acute malnutrition—illnesses, vaccinations, household education and access to safe water, in particular—to enable fine-grained predictions of household risk.

## 3 Materials and methods

### 3.1 *D2A* app

The survey questionnaire was developed in XLSForm, a standard for building Excel forms and implemented through the non-profit *KoboToolbox* (44, 45). The resulting *D2A* questionnaire, activated by scanning a QR code, was deployed using *KoboCollect* (v2022.1.2). *KoboCollect* is available for Android smartphones and can be installed directly via the Google Play Store. The app allows respondents to enter unlimited survey submissions, including photos and voice messages. Whereas an internet connection is not required for data entry, surveys are transferred to the server automatically once devices reconnect to a network.

**Figure 1:**
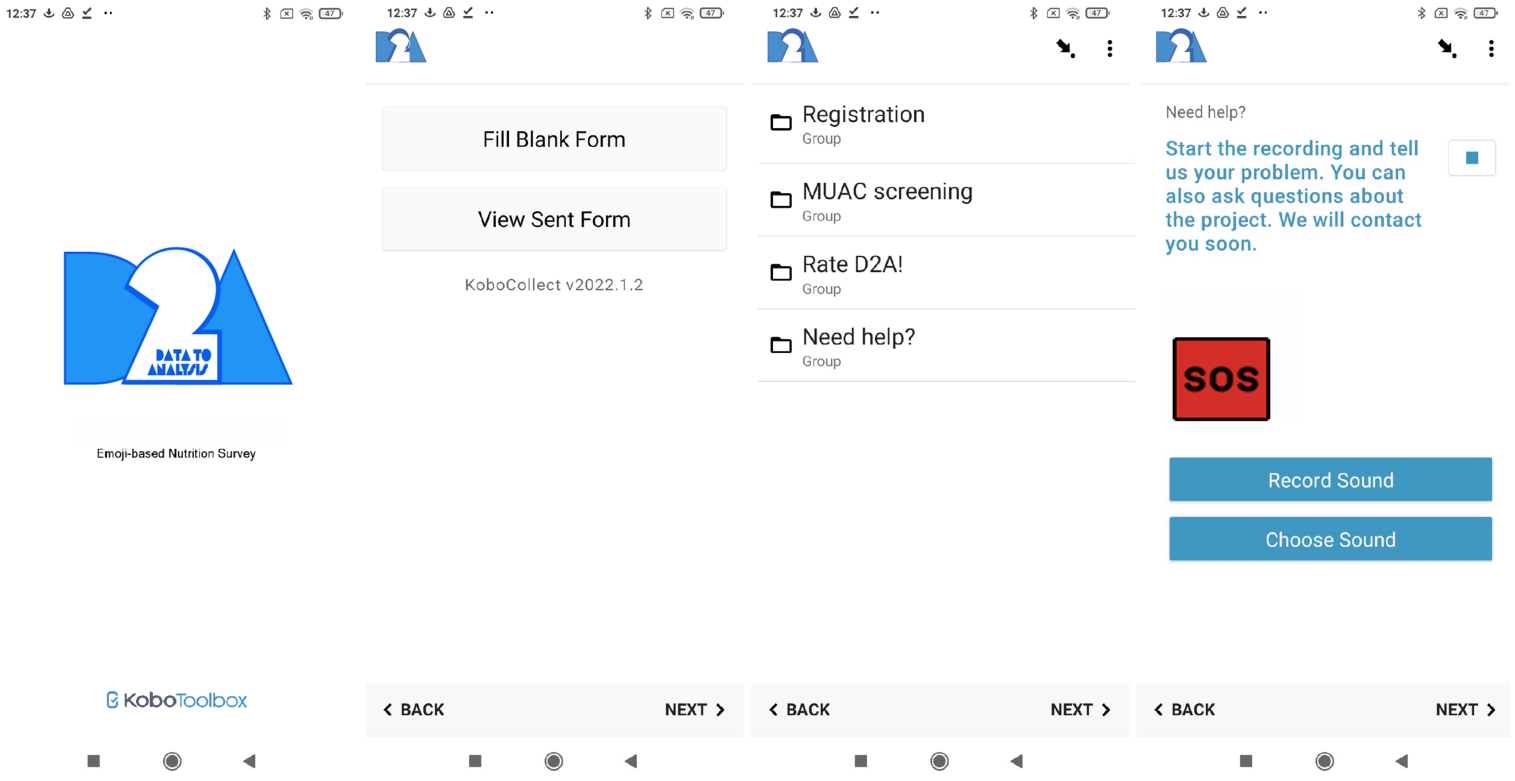
*D2A* loading screen, landing page, survey selection menu and help screen **(from left to right)**. All emojis designed by OpenMoji - the open-source emoji and icon project. License: CC BY-SA 4.0.

Figure 1 illustrates the *D2A* loading screen (left side) and landing page (mid-left) displayed after opening the *KoboCollect* application. The survey selection menu on the mid-right side in Figure 1 displays four different questionnaires users were able to select at various data collection stages. Access for first-time users is restricted to the registration survey. The registration questionnaire disappears upon successful completion and users are then able to carry out and report Family MUAC screenings. The “*Need help?*–screen,” as illustrated on the right side of Figure 1, enables users to record and submit a voice message to ask questions, describe problems, and request further assistance.

**Figure 2:**
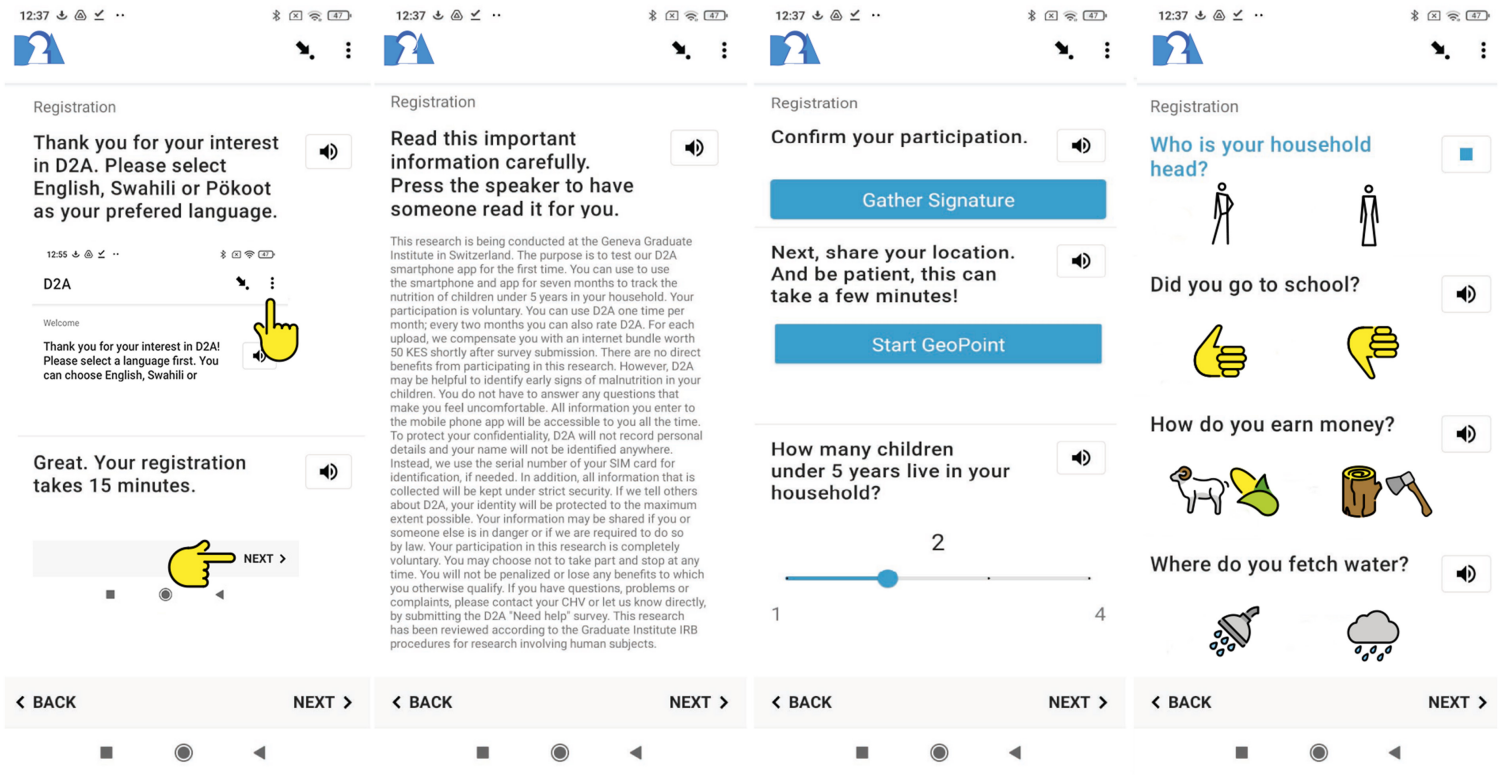
*D2A* registration survey. All emojis designed by OpenMoji - the open-source emoji and icon project. License: CC BY-SA 4.0.

**Figure 3:**
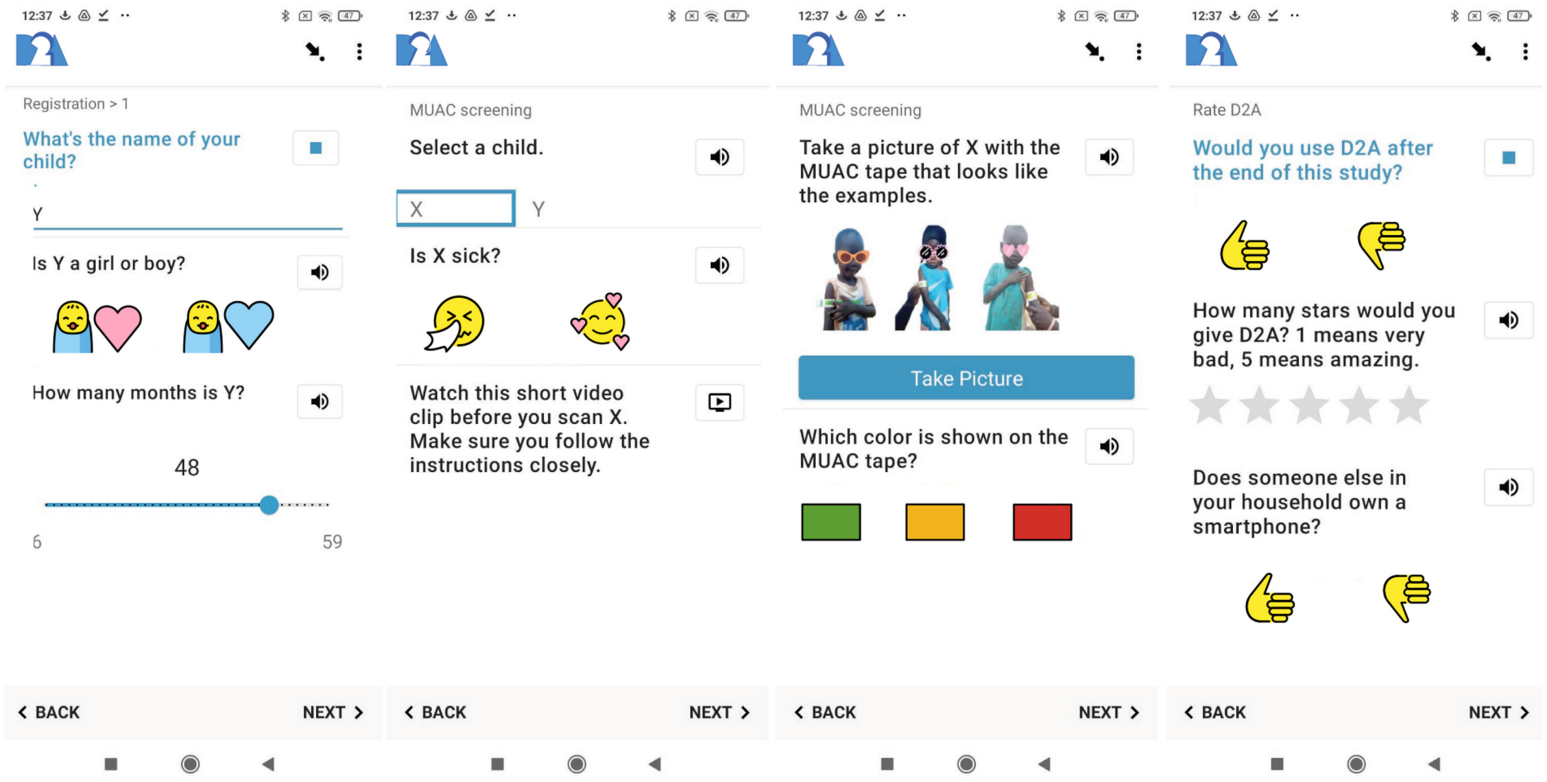
*D2A* registration survey, MUAC screening, and feedback survey **(from left to right)**. All emojis designed by OpenMoji - the open-source emoji and icon project. License: CC BY-SA 4.0.

Figure 2 on the left displays the registration survey. Participants are identified based on their SIM card serial numbers. This information is registered automatically by *D2A*, and no family names are required. Questionnaires are translated to English, Swahili and Pökoot, the local dialect spoken in West Pokot. Users can select their preferred language and familiarize themselves with the swiping-logic in the app (left side). Given that a key barrier to participation is widespread illiteracy, survey questions and project information can also be played as an audio-recording, by pushing the speaker-icon. After users consent to participate in the study by clicking the “*Gather Signature*-button” (mid-right), *D2A* registers the current location of users. Next, users enter a few household characteristics by selecting emojis designed to be culturally appropriate, and validated in a pilot study. To complete registration, users are asked to specify the name, gender, and age for each child under five years of age in their household, as illustrated on the left side in Figure 3. The number of repetitions of this group of questions varies with the number of children specified earlier in the survey. The aim of the registration survey for first-time users is to keep the information required in the monthly MUAC screenings to a minimum. The MUAC screening is depicted on the mid-left and mid-right in Figure 3. To encourage complete responses, users are prompted to measure all registered children every time they use *D2A*. As illustrated on the mid-left, users can select a child from the list of names provided during the registration. After the mandatory 90-second video clip with Family MUAC instructions to remind participants of some of the key points in the measurement protocol, for instance the correct arm and how the exact place for measurement is determined, users can carry out the screening. To complete the survey, participants are asked to take a picture of their child with the MUAC tape following the examples provided and select the color on the Family MUAC tape, as shown in Figure 3 on the mid-right. Figure 3 on the right depicts the feedback survey that users can submit once every two months.

### 3.2 Pilot study objectives

The study aims to test household-led nutrition screenings via the *D2A* smartphone app. The term “household-led” implies that any adult in the home—a mother, father, older sibling, or caregiver—is eligible to conduct nutrition screenings via the smartphone app, as long as they are familiar with the Family MUAC methodology and indicate their role at the beginning of each nutrition screening. We consider three key dimensions. First, the *accuracy* of Family MUAC observations tracked via the *D2A* smartphone app relative to paper-based Family MUAC screenings. Second, the *acceptance* of self-reporting over time and across livelihood contexts, given the additional responsibility placed on those most vulnerable. A related concern pertains to household *participation* rates in the presence/absence of trained CHVs—whether their presence significantly impacts dropout. And third, the *cost* of household-led nutrition screenings via the *D2A* smartphone app relative to conventional paper-based methods employed by the NDMA on a monthly basis. In particular, we compare the high upfront costs of smartphones to the monthly personnel and travel costs of NDMA enumerators per household over a 12-month period.

### 3.3 Empirical context

Household-led Family MUAC reporting via the *D2A* smartphone app was tested in West Pokot County, Kenya, between July, 2022 and January, 2023. The cumulative effect of five failed rainy seasons (46) and a cost of living crisis, exacerbated by the war in Ukraine (11, 47), diminished the purchasing power of many households in West Pokot (48) even before a full recovery from COVID-19 income losses was possible (49). In addition, years of drought worsened chronic stressors—limited access to safe drinking water, poor WASH (Water Sanitation and Hygiene) practices, and increased risk of disease (50). Livestock pests, reduced milk production, and poor feeding practices further hindered disease recovery, as did a deterioration in health care services with rising numbers of admitted children (51).

Historically, “*Family MUAC* screenings” (52, 53) were conducted face-to-face with the aid of a color-coded MUAC tape. Despite the “Family” qualifier, the primary responsibility of measurement fell on field monitors or CHVs trained to use the tape correctly and maximize data quality, given that small inaccuracies—literally millimeters—significantly impact validity. When mobility was constrained due to COVID-19 regulations, however, many Kenyan households in arid and semi-arid lands (ASAL) were trained to carry out the screenings themselves. As a result, most households are now capable of diagnosing their children for acute malnutrition, and simple color-coded MUAC tapes are readily available (52). Yet, results from these screenings are typically unreported, beyond their use for immediate diagnosis.

### 3.4 Sampling and participants

The *D2A* smartphone app was tested in conjunction with the National Drought Management Authority (NDMA) county office. The NDMA of Kenya is a governmental organization tasked with drought and nutrition monitoring in 23 ASAL counties. To inform monthly nutrition risk assessments, each of the 23 NDMA county offices deploys enumerators to track monthly anthropometric information of some 150 children alongside key household-level covariates. In four to five villages, the same households are sampled over a period of 12 consecutive months. NDMA's pen-and-paper surveys constitute the gold standard in terms of geographical and temporal resolution in nutrition monitoring projects across sub-Saharan Africa and are used as a benchmark against which we assess the accuracy and cost of data collection with our smartphone app.

**Figure 4:**
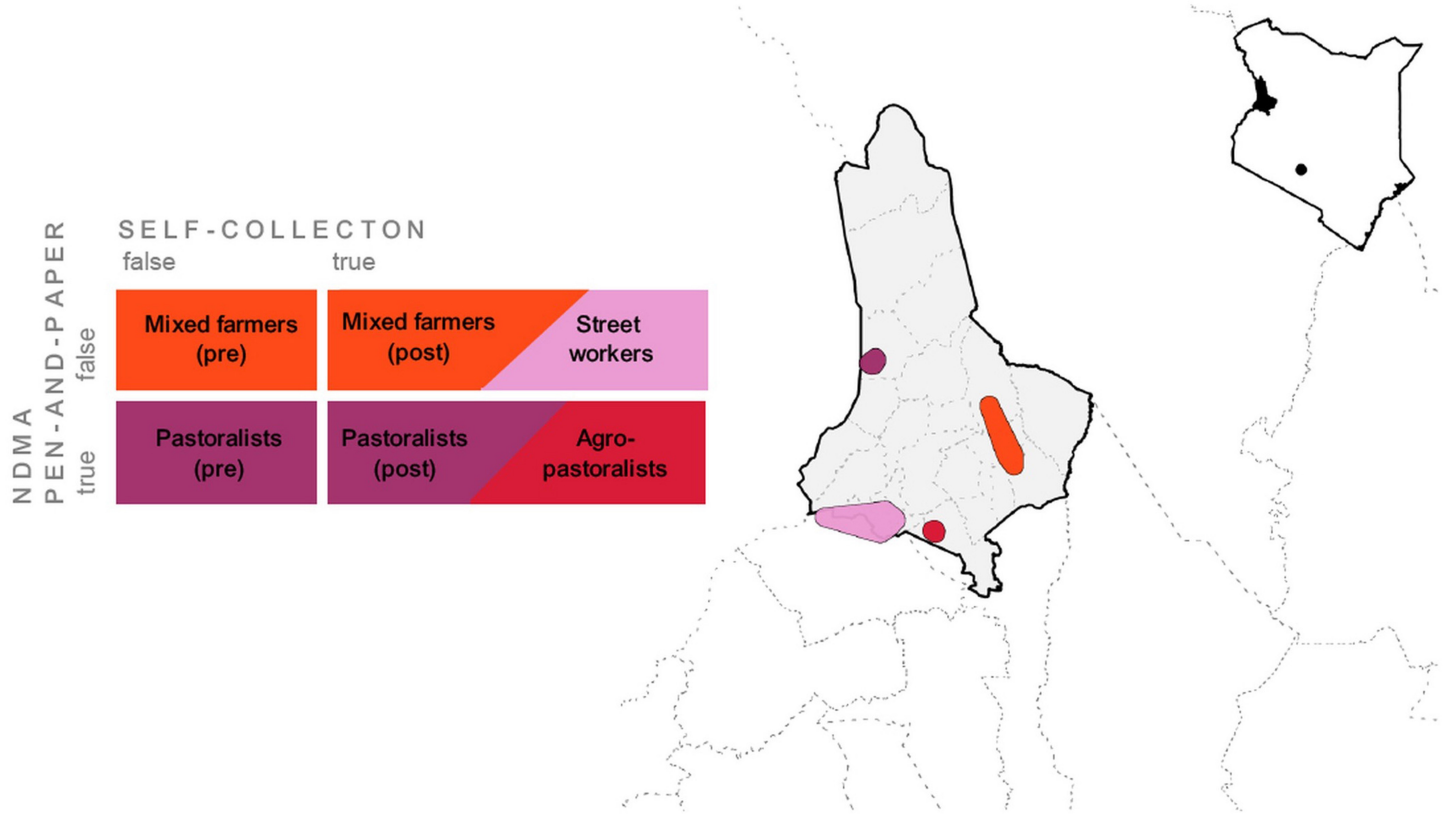
Map of West Pokot County, Kenya, with the location of four communities and their assignment to treatment groups. A 5,000 meter buffer was added around precise locations of households.

Effective implementation of the pilot study, in collaboration with the NDMA, required the prioritization of NDMA preferences and needs over random assignment and internal validity. For the benchmark comparison of self-reported smartphone surveys and pen-and-paper methods, we selected two communities that were also sampled in monthly NDMA nutrition risk assessments. As NDMA nutrition tracking is limited to agro-pastoralist and pastoralist regions, we selected one community for each livelihood. To test the feasibility of self-reporting across a wider range of contexts, we selected two non-NDMA sites, in particular a group of mixed farmers and street workers. The study design is summarized in Figure 4. The number of participating households was determined by available funds for smartphones and overall feasibility. To ensure sufficient variation across communities and treatments while maintaining a reasonable sample size, 30 volunteer households were sampled in each group after a brief in-person introductory session in mother support groups organized by CHVs.

**Figure 5:**
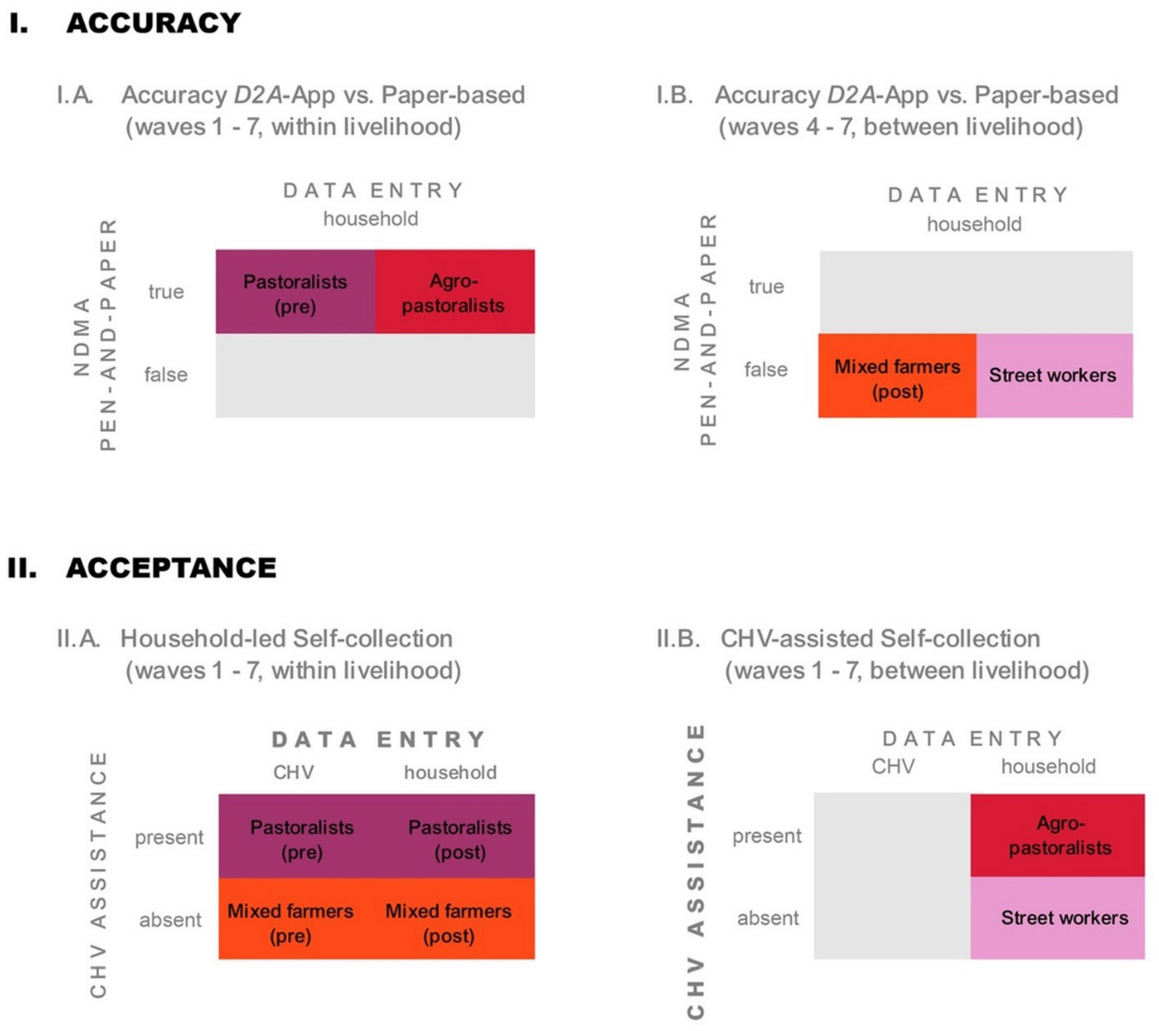
Overview of quantitative group comparisons and corresponding sub-samples of data. **(I.A)** Accuracy of *D2A*-App vs. paper-based Family MUAC screenings, within livelihood. **(I.B)** Accuracy of *D2A*-App vs. paper-based Family MUAC screenings, between livelihoods. **(II.A)** Acceptance of data entry by CHVs vs. self-collection, within livelihoods. **(II.B)** Acceptance of CHV-assisted self-collection vs. those with no CHV assistance, between livelihoods. *Self-collection* indicates whether MUAC data were entered by households or CHVs, and *CHV present* whether a CHV supported households during data collection.

By covering the top-four livelihoods in West Pokot, we were able to assess the scalability of the pilot and generate insights on effective participant recruitment strategies. By covering communities with and without the presence of NDMA CHVs, we also gained valuable insights on survey administration procedures and intervention design. More specifically, we assigned two “control communities”—pastoralists and mixed farmers—where monthly Family MUAC data was *not* self-collected by households at the outset, but entered into the app by CHVs during monthly household visits (*Pastoralists (“pre”)* and *Mixed farmers (“pre”)*). As illustrated in Figure 4, these two communities switched to household-led self-collection in October 2022 (*Pastoralists (“post”)* and *Mixed farmers (“post”)*), after survey data were entered into *D2A* by project staff for four consecutive months. In addition, two “self-collection communities”—agro-pastoralists and street workers—self-reported Family MUAC data via *D2A* over the entire seven-month period. In these communities, some households were supported by an NDMA CHV, while others received no CHV support. We thus use two main indicators to describe the study design: (*i*) *self-collection* = *true/false*, indicating whether MUAC data were entered by households (*true*) or by CHVs (*false*), and (*ii*) *CHV present* = *present/absent*, indicating whether a CHV assisted households during data collection. This design allows for within- and between treatment comparisons of three groups, later referred to as self-collection = *false [“pre”]*, self-collection = *true [“post”]*, and self-collection = *false [“true”]*. The latter refers to the agro-pastoralist and street worker groups, where households self-collected data over seven months, either in presence or absence of a CHV. Figure 5 overviews the resulting sub-samples of data used for different group comparisons in the quantitative analysis. Supplementary Section I provides additional details on the sampling.

### 3.5 Training and data collection

Participants were first trained to carry out the Family MUAC screening and introduced to the basic features and functionalities of smartphones—including the built-in torchlight and access to the internet. We explained that smartphones were provided free of charge for the duration of the study, and that SIM cards could be registered with our assistance. SIM card (not phones) could be retained by households if they dropped out of the study, or when the study came to an end. One hundred and twenty households received a SIM card and a *Xiaomi Redmi 9C* smartphone with 32GB memory and 2GB RAM approximately one week later, in a second training on the *D2A* app. In this session, households completed the registration survey and comprehension checks were conducted to make sure the main functions and survey items in the app were understood correctly by all participants. Before the end of the first data collection cycle, project staff followed-up with each household to clarify problems and questions individually. Upon completion of each monthly reporting cycle, participants were compensated with an internet bundle worth 50 KES (or 50 KES in cash if the data was collected by a field monitor).^1^ A field manual with Family MUAC guidelines and other useful information, for instance a timeline and tasks for the seven-month data collection period (see Supplementary Figure S1) or a “*D2A*-cheat sheet” (see Supplementary Figure S2), was handed out to all participants, project staff, and CHVs.

### 3.6 Data governance and privacy

Survey forms and responses were stored on servers hosted by Amazon Web Services. All identifying information was automatically replaced with pseudonymized device-based identifiers and server access was restricted to key project staff through role-based controls. All data-handling procedures were reviewed and approved by ethics committees in Kenya (AMREF ESRC) and abroad, ensuring compliance with both national and international data-protection norms, in particular the General Data Protection Regulation (GDPR) and Kenya's Data Protection Act (2019).

### 3.7 Quantitative analysis

#### 3.7.1 Accuracy

The sample collected via the *D2A* app comprises 920 monthly Family MUAC observations of individual children from 180 households, measured repeatedly over a maximum of seven data collection cycles. Summary statistics are presented in Supplementary Table S1. To explore the effectiveness of smartphone nutrition surveys relative to conventional pen-and-paper methods, we conduct a paired comparison of classification accuracy in self-reported Family MUAC measures via the *D2A* app and pen-and-paper surveys, *within* the same pastoralist and agro-pastoralist communities and relative to the West Pokot baseline. Both surveys were administered simultaneously, albeit with non-overlapping participating households and hence different children. To account for the fact that the same children were not sampled in both surveys, children are matched by month, community, and additional household characteristics. For small-N designs like ours, the matching algorithm selects the largest sub-sample of exact matches. As a function of larger numbers of Family MUAC observations in the pen-and-paper survey, we initially faced a trade-off between bias due to imbalance (keeping self-collected observations but not finding good matches in the pen-and-paper survey) and bias due to incomplete matching (54) (discarding self-collected observations to find good matches in the pen-and-paper survey).

Hence, we use 1:1 cardinality matching (55, 56) on the ATT. Cardinality matching identifies the largest subset of observations for which balance for livelihoods, months and selected household-level key drivers and baseline conditions of acute malnutrition (female-headed households, unsafe water sources, and education), is achieved given a 0.05 threshold. By dropping a small number of observations, we are able to reduce noise in the data and obtain the robust estimates of reported effects. Supplementary Figure S3 illustrates the corresponding balance plot with absolute standardized differences in means for the above-mentioned baseline conditions and household-level predictors of acute malnutrition, before and after 1:1 cardinality matching of monthly Family MUAC observations collected via the *D2A* app and pen-and-paper surveys. Supplementary Table S2 summarizes additional balance metrics for the adjusted and unadjusted samples.

The matched sample features 147 individual Family MUAC observations that were self-collected with the *D2A* app and 147 more paper-based surveys collected simultaneously in the same communities. The target against which each sub-sample is assessed in terms of classification accuracy includes all remaining Family MUAC observations in West Pokot sampled by the NDMA. For comparability, all paired accuracy analyses use down-sampled target data with matched sample sizes while preserving MUAC category distributions. Supplementary Table S3 provides matched summary statistics grouped by survey, and the West Pokot target. For each matched observation, we compare the MUAC classification recorded in *D2A* or the paper-based survey against the corresponding class in the West Pokot target. We distinguish three binary Family MUAC classification tasks: *not green* (MUAC < 125mm), *yellow* (corresponding to Moderate Acute Malnutrition [MAM], MUAC 115mm–125mm), and *red* (corresponding to Severe Acute Malnutrition [SAM], MUAC < 115mm) (4).

We obtain a confusion matrix with true positive (TP), true negative (TN), false positive (FP), and false negative (FN) classifications for each task and both smartphone- and paper-based data collection tools. Sensitivity, defined as the proportion of “positive” observations relative to the total number of malnourished in the West Pokot target, is calculated as TP/(TP + FN). The proportion of negative observations relative to the total number not malnourished in the target population is calculated as TN/(TN + FP) and referred to as specificity. McNemar's test is then used to assess whether paired differences in accuracy between *D2A* and paper-based classifications are statistically significant (57, 58). Matching and balance plotting is performed in R, using the *MatchIt* (59, 60) and *cobald* (61) packages. The *DTComPair* R-package (58) is used for the paired comparison of classification accuracy (57).

#### 3.7.2 Acceptance

To assess the effects of self-collection by households in the presence/absence of CHVs on study completion rates, missing household and child characteristics other than the Family MUAC are imputed using a simple “last observation carried forward (LOCF)” approach in the *imputeTS* R-package (62). Complete observations are then aggregated to the household-level. We used the mode for fixed household characteristics and calculated study completion rates for each household relative to the largest possible number of survey waves, depending on the treatment group, i.e., 7 (self-collection = *false [“true”]*) or 4 (self-collection = *false [“pre”]* and self-collection = *true [“post”]*). Supplementary Table S4 provides aggregated summary statistics.

#### 3.7.3 Relative cost

Information provided by the NDMA West Pokot County office on the monthly expenditures for their nutrition screenings in West Pokot is used to calculate the relative monthly cost for one household screening. This amount is compared to the app-based nutrition screening cost (which includes smartphone, mobile data bundles, and CHV daily rates for training and follow-up visits) per household, adjusted to the 12-month sampling period used by the NDMA.

### 3.8 Qualitative analysis

In the early stages of the pilot study, we interviewed international and local experts in the field of nutrition to evaluate the app-based questionnaire, app design features, and anticipate potential challenges, prior to testing *D2A* with NDMA nutritionists, enumerators, and local CHVs (see Supplementary Table S5 for the interview questionnaire). Given the small body of empirical evidence on household-led nutrition screenings and reporting via smartphones, we also conducted periodic Focus-Group-Discussions (FGD) with study participants to complement our quantitative analyses. These discussions pertained to app design features and additional functionalities, as well as user preferences regarding nutrition screening modes and reasons for dropout (see Supplementary Table S6 for a list of topics covered in the FGDs). Once the data were collected, local experts were consulted for ground-truthing and validation.

## 4 Results

We analyzed the *D2A* smartphone app along three key dimensions—accuracy, acceptance, and cost—relative to paper-based nutrition surveillance tools.

### 4.1 Accuracy

#### 4.1.1 Measurement validity

**Figure 6:**
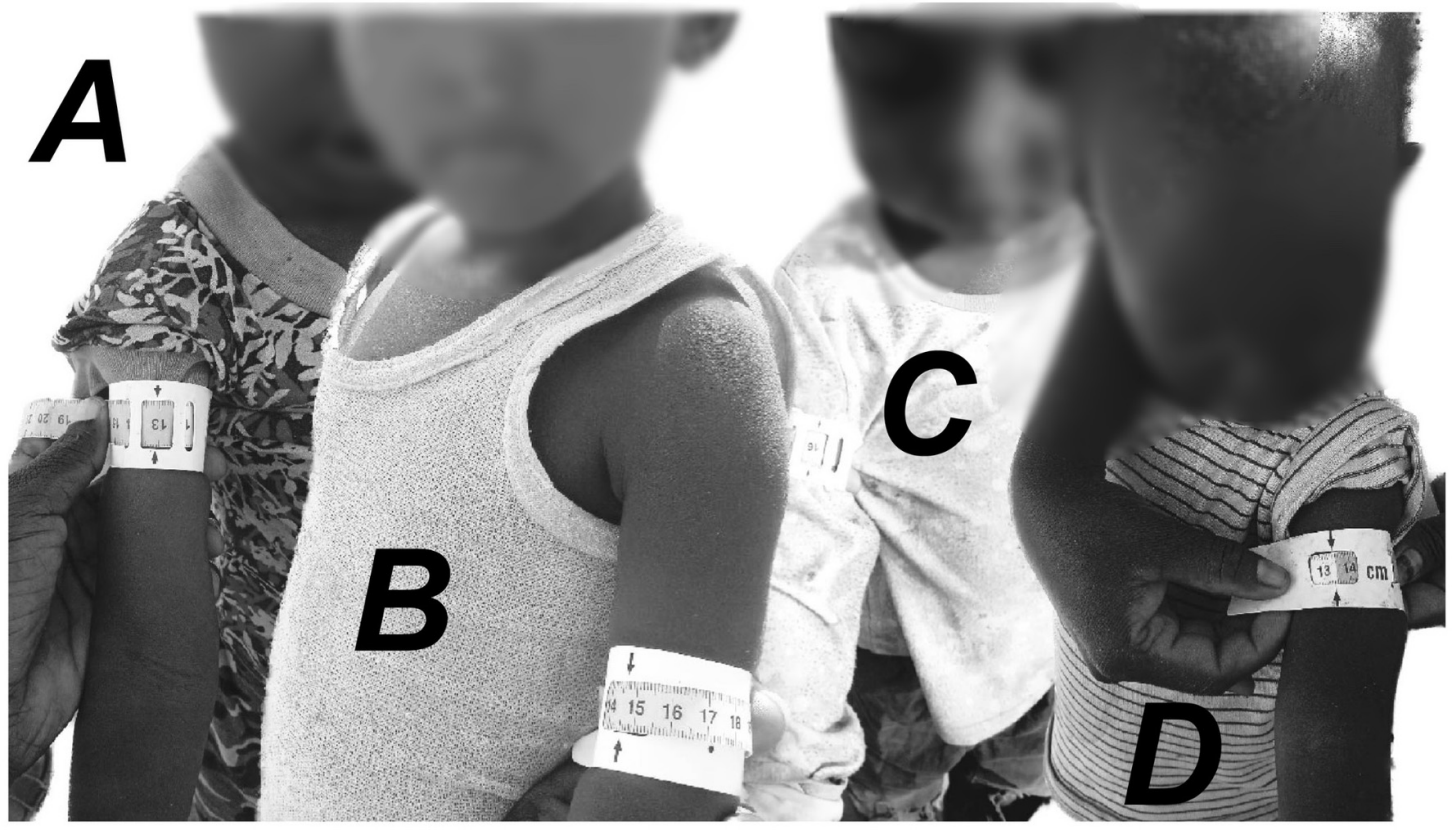
Examples of invalid Family MUAC measures, i.e., Family MUAC tape placed too far up **(*A*)** or down **(*B*)**, above a shirt **(*C*)** or too tight Family MUAC tape **(*D*)**.

We reviewed all pictures of children with MUAC tapes uploaded by households to assess measurement validity. Responses were dropped if a child was not photographed according to the examples provided directly in the survey. Figure 6 exemplifies additional Family MUAC measurement issues that led to the exclusion of observations from the sample; specifically, if the Family MUAC tape was placed too far up (*A*) or down (*B*), above a shirt (*C*) or if the Family MUAC tape was too tight (*D*). Out of the 974 Family MUAC measures obtained in this study, 54 observations (5.5%), missing at random across treatment groups, were excluded. In addition, responses for certain survey items were excluded if they were unclear or appeared false—for instance, if the child's age did not correspond to the age of the child in a picture. We note two covariates with missing observations: child age (12%) and the number of children in a household (11%). Supplementary Figure S4 provides an overview of the missing value rates for each covariate in the raw data. The overall rate of 1.6% in the *D2A* sample is lower relative to the missing value rate for paper-based surveys—11.4% in the pen-and-paper sample including all observations in West Pokot between July 2022 and January 2023.

#### 4.1.2 Benchmark comparison

**Figure 7:**
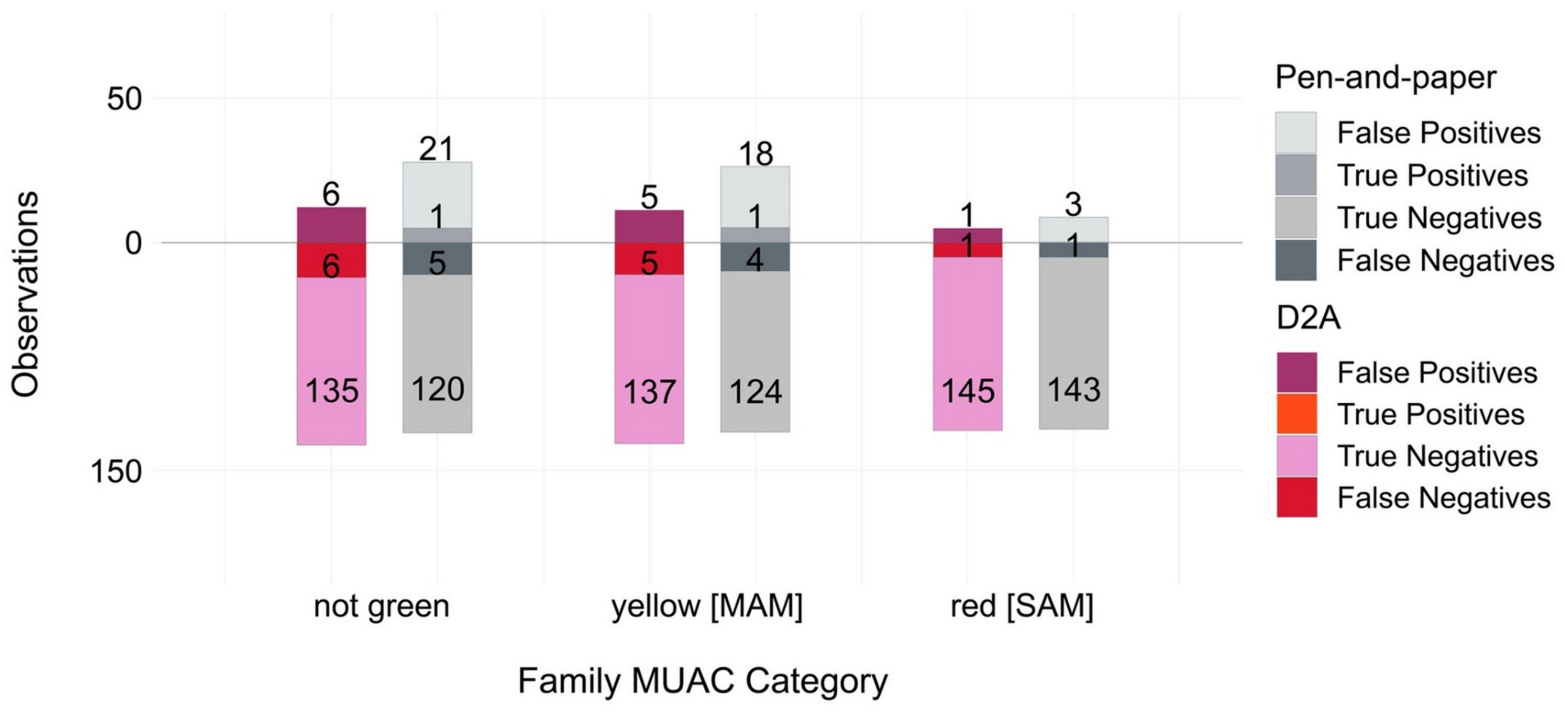
Confusion matrices for three Family MUAC categories in the *D2A* app and paper-based survey, relative to the West Pokot target. The matched sample includes *N* = 441 Family MUAC observations with *N* = 147 observations for the app- and paper-based sample and the West Pokot target.

Figure 7 summarizes the confusion matrices for the three MUAC categories. Two general trends are reflected in this comparison. First, the majority of children in our sample are healthy insofar as they did not test “positive” for acute malnutrition. And second, discrepancies between smartphone and paper-based classifications are minimal, regardless of classification tasks.

The confusion matrices depicted in Figure 7 constitute the basis for the paired accuracy metrics reported in Supplementary Table S8. Table 1 compares the *sensitivity* and *specificity* of observations collected via the *D2A* smartphone app and pen-and-paper surveys for “*not green*,” *yellow* [MAM], and *red* [SAM] classifications, using the remaining sample of observations collected during our study period in West Pokot County as the target. While McNemar's test reveals no significant differences in sensitivity regardless of Family MUAC classifications, we found small, albeit significant differences (*p* < 0.01) in specificity for “*not green*” (δ = −0.106, 95% CI = −0.157, –0.055) and *yellow* [MAM] (δ = −0.092, 95% CI = −0.139, –0.044) categories. Negative specificity estimates point to the more accurate detection of true negative observations, in particular for the “*not green*” category, via the smartphone app.

**Table 1:** Paired comparison of classification accuracy in self-collected Family MUAC measures via the *D2A* app and pen-and-paper surveys, *within* livelihood groups and relative to the West Pokot baseline.

	**Family MUAC**	***D2A* App**	**Pen-and-paper**	**Diff**.	**Diff. SE**	**95% CI_*Lower*_**	**95% CI_*Upper*_**
Sensitivity	“Not green”	0	0.167	0.167	0.152	−0.132	0.465
	Yellow [*MAM*]	0	0.2	0.2	0.179	−0.151	0.551
	Red [*SAM*]	0	0	0	0	0	0
Specificity	“Not green”	0.957	0.851	−0.106^*a*^	0.026	−0.157	−0.055
	Yellow [*MAM*]	0.965	0.873	−0.092^*a*^	0.024	−0.139	−0.044
	Red [*SAM*]	0.993	0.979	−0.014	0.01	−0.033	0.005

Self-collected Family MUAC measures via the *D2A* app (self-collection = *true* and self-collection = *true (“post”)*) in the NDMA sites (agro-pastoralist and pastoralist communities), were compared to pen-and-paper survey data from different children in the same communities. Observations were first matched and paired using 1:1 cardinality matching on the ATT. Next, observations in the West Pokot baseline for the same timeframe were down-sampled to *N* = 147, corresponding to the sample size of the matched and paired *D2A* and pen-and-paper sub-samples while preserving the probability distribution of Family MUAC categories. McNemar's test was used to assess significance of differences in sensitivity and specificity of both survey types using the West Pokot baseline as target. ^*a*^, *p* < 0.01.

Focusing on internal validity, the matched and paired analysis for households with the same livelihoods allows us to assess the classification accuracy for self-collected Family MUAC data relative to pen-and-paper surveys. With a complementary focus on external validity, the between-group analysis presented in Supplementary Section II provides insights on the feasibility of self-collection across a wider range of livelihood contexts. Supplementary Figure S4 and Supplementary Table S9 illustrate the confusion matrix and overview accuracy metrics. The between-livelihood comparison yields small, insignificant differences in sensitivity and specificity of smartphone and pen-and-paper Family MUAC classifications, using West Pokot as a target. Overall, the findings of the sensitivity analysis indicate that self-reported nutrition surveys across a wider range of households are feasible, insofar as this generates the same accuracy as data collected by means of pen-and-paper. Furthermore, the slightly lower specificity of pen-and-paper surveys reported in the within-group analysis is visible (but not significant) in the between-group comparison, providing further evidence for higher accuracy of Family MUAC observations collected via the smartphone app.

### 4.2 Acceptance

#### 4.2.1 Participant dropout

**Figure 8:**
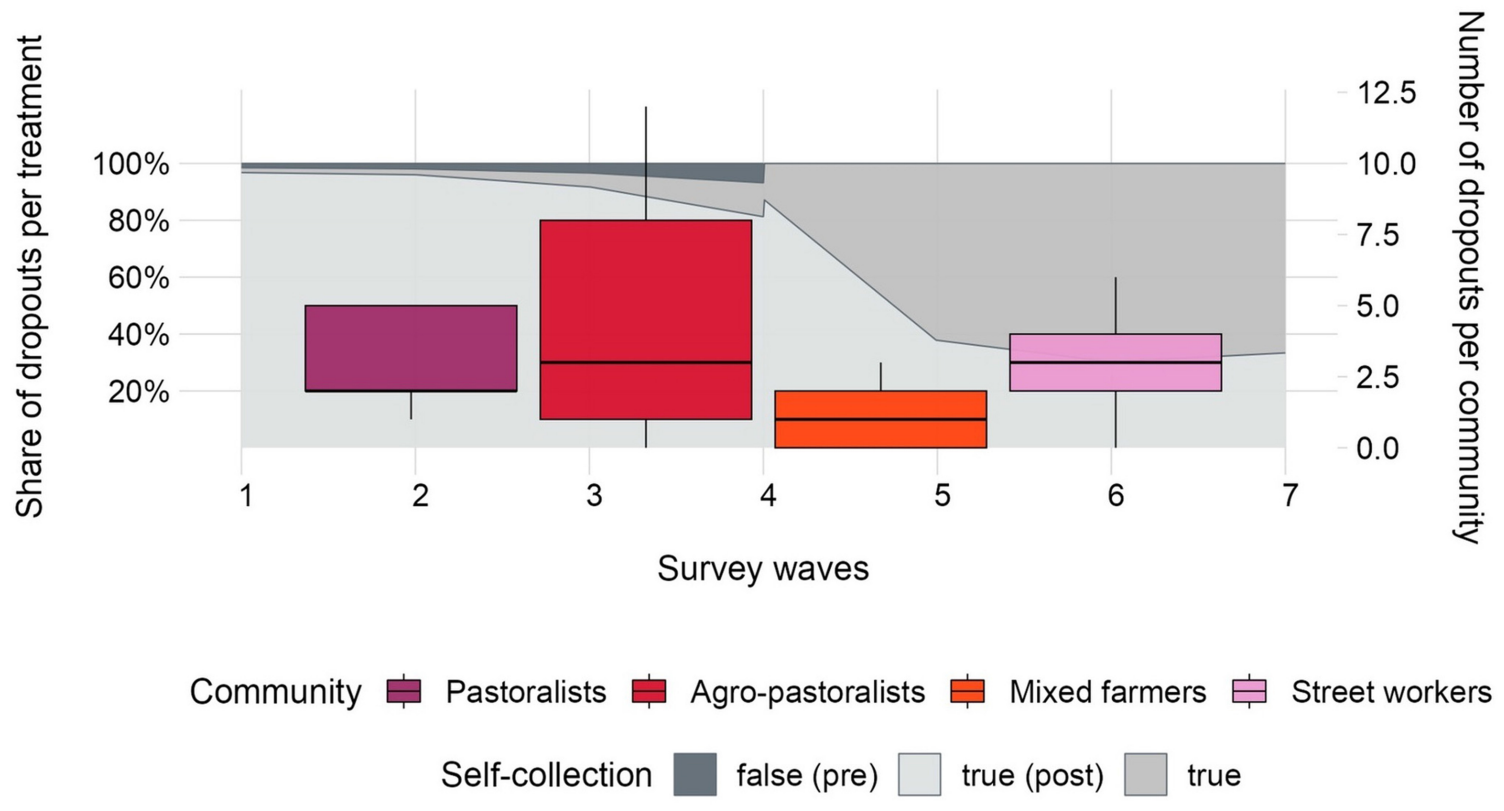
Number of monthly dropouts from *N* = 180 households by livelihood (red/pink boxplots), and percentage of monthly dropouts per treatment group (gray area plot).

Figure 8 illustrates the distribution of household dropout over monthly survey waves per treatment and livelihood group. As expected, the share of dropouts in the “control” group (self-collection = *false*), was marginal given monthly follow-ups by field monitors, while the share of dropouts in the pastoralist and street workers communities (self-collection = *true*), increased with higher survey waves (after four rounds of data collection). In general, we note the high acceptance of our pilot across livelihoods, with lowest dropout rates in the pastoralist (less than 2 monthly dropouts) and mixed farmer communities where on average one participant dropped out after each survey wave. Bonferroni-adjusted *t*-tests, as summarized in Supplementary Table S10, indicate significant differences in dropout between the mixed farmer community and all other livelihood groups. As illustrated in Figure 8, monthly dropouts in the agro-pastoralist and street worker communities were very similar with an average of three participants, with the agro-pastoralist group displaying the largest range between survey waves and maximum number of eleven dropouts in 1 month.

#### 4.2.2 Household self-reporting

**Figure 9:**
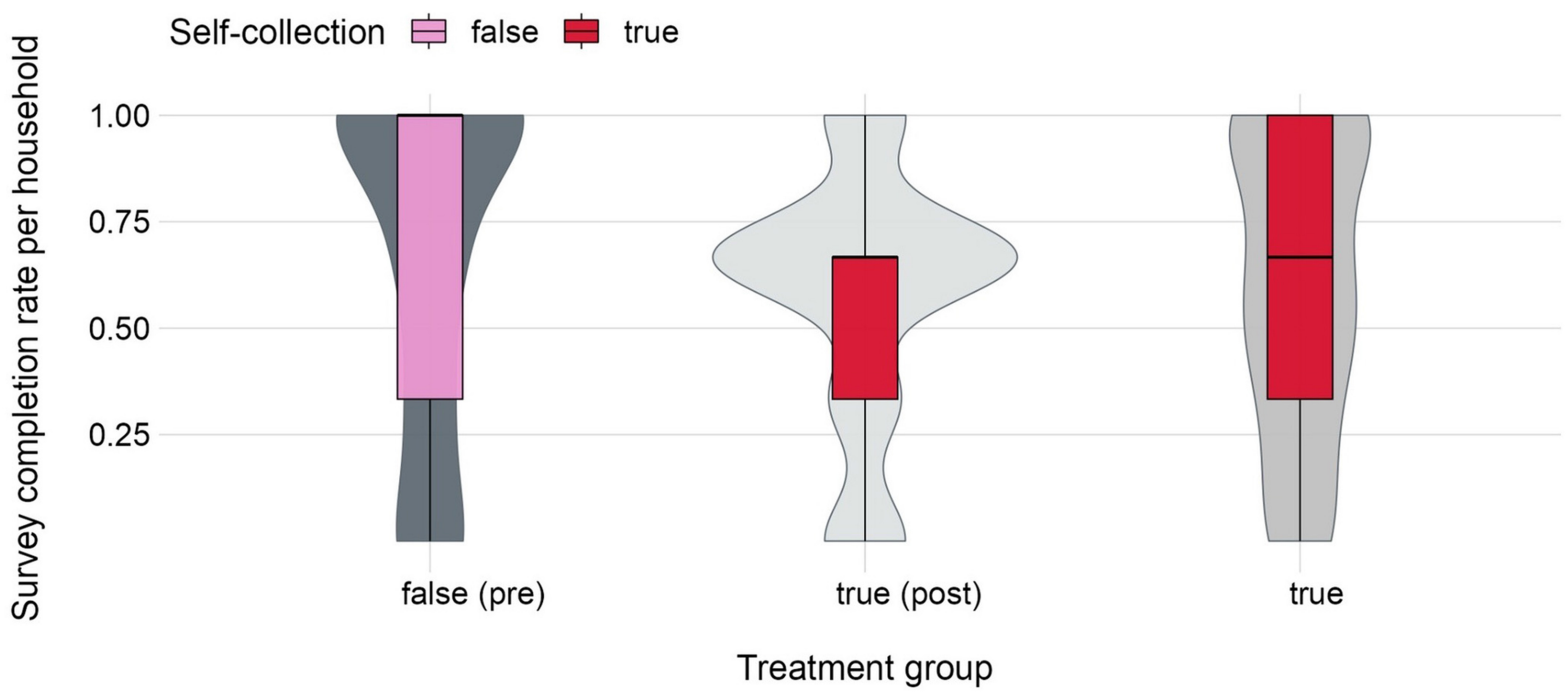
Distribution of survey completion rates from *N* = 180 households when data was self-collected by participants relative to when CHVs entered responses. Violin plots are depicted in gray and show completion rates per treatment group. Boxplots are depicted in red/pink and show completion rates depending on whether data was self-collected by households or entered into the *D2A* app by CHVs.

Next, we explore survey completion rates when data was self-collected by mothers and caregivers relative to when CHVs entered responses into the *D2A* app. Figure 9 illustrates the distribution of survey completion rates per household in the three treatment groups, depending on whether data was self-collected or not. While survey completion rates were highest in the control group (self-collection = *false [“pre”]*), Paired *t*-tests indicate no significant differences in average household completion rates for either between group comparison (see Supplementary Table S11).

#### 4.2.3 CHV-assisted self-reporting

**Figure 10:**
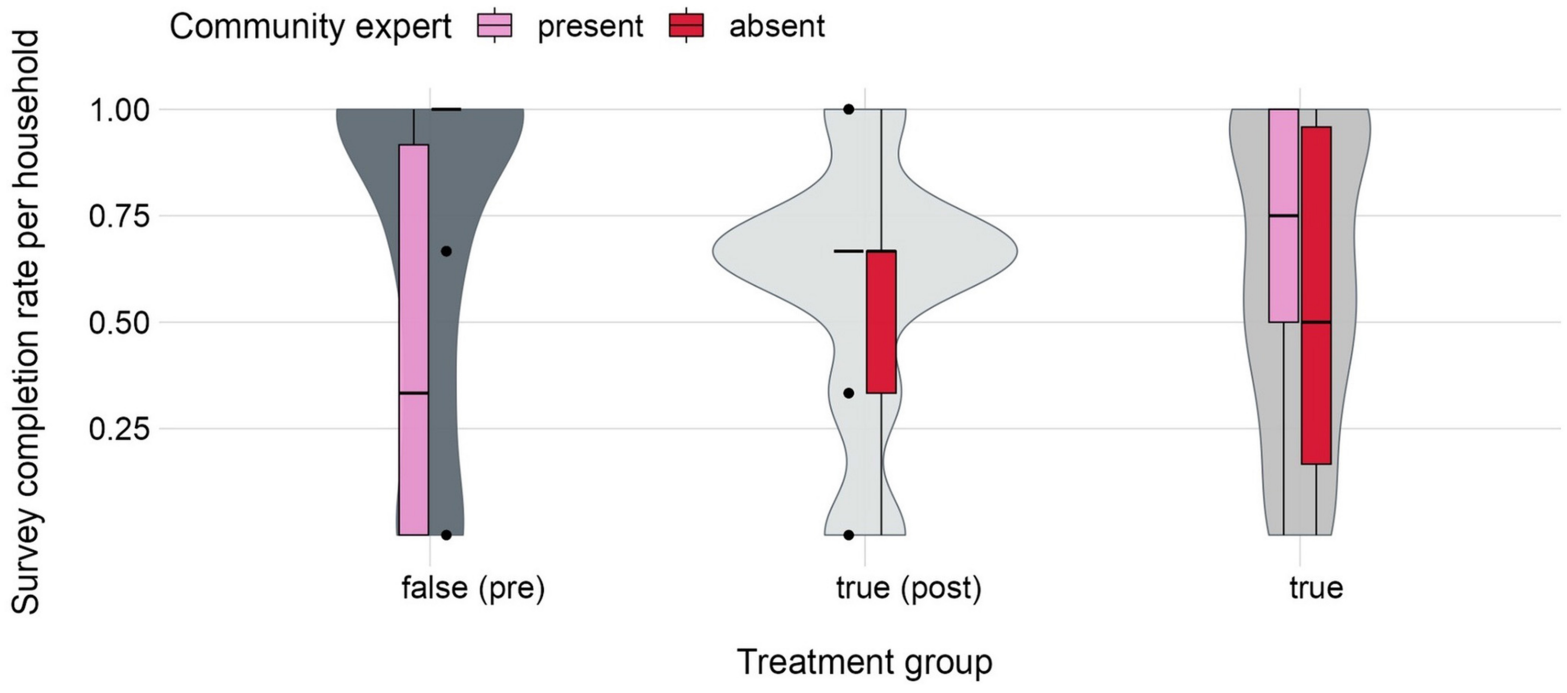
Distribution of survey completion rates from *N* = 180 households depending on the presence or absence of a CHV. Violin plots are depicted in gray and show completion rates per treatment group. Boxplots in red/pink show completion rates depending on whether a trained CHV was present or absent.

Figure 10 reflects the survey completion rates per treatment group depending on the presence or absence of a CHV. If data was not self-collected with the *D2A* app (treatment group = *false [“pre”]*), a within group comparison yields that survey completion rates were significantly higher in the absence of an NDMA CHV (paired *t*-test: *t* = −4.644, df = 29, *p* = 0.0004). If data was self-collected with the *D2A* app, a between-group comparison indicates that survey completion rates were significantly lower in the absence of a CHV (paired *t-*test: *t* = 2.6722, df = 59, *p* = 0.009). In particular, groups with a CHV have 15% higher survey completion rates relative to groups with no CHV present (mean diff. = 0.152, 95% CI = 0.038, 0.267).

### 4.3 Relative cost

Monthly nutrition surveys administered by the NDMA over 12 consecutive months are unparalleled in the region. Yet, high-frequency face-to-face surveys are time- and labor-intensive, especially if training and travel costs for enumerators are taken into account. According to NDMA West Pokot County staff, administering the paper-based nutrition screening in one household costs approximately 1,585 KES (approx. 12.25 USD) per month, and 19,020 KES (approx. 147 USD) over 12 monthly data collection cycles. The acquisition cost per smartphone used in our study was 10,500 KES (approximately 88 USD in 2024 terms). Adding 12-month data bundles for each participant, CHV salaries and daily rates for two initial training sessions and one monthly follow-up visit to each household, brings the total cost per household in our study to 12,500 KES (approx. 97 USD). Compared to 19,020 KES per household and year for the paper-based nutrition screening conducted by the NDMA, we find that self-collection of Family MUAC by households for the same period of time is significantly less costly.

## 5 Discussion

Accurate and reliable data, reported in near-real time and robust to problems of accessibility, is necessary for the detection and timely treatment of child acute malnutrition. Our pilot demonstrates the potential of low-cost acute malnutrition assessments via smartphone apps by exploring acceptance, accuracy, and cost of self-collection relative to conventional face-to-face nutrition screenings.

The first theme concerns diagnostic accuracy. Our findings are consistent with previous work showing that mothers and caregivers can obtain Family MUAC measurements with high accuracy (19, 63, 64). The fact that sensitivity and specificity closely matched those of pen-and-paper assessments suggests that shifting routine MUAC collection to caregivers does not compromise data quality. For health systems, this is a critical insight: decentralizing measurements while retaining diagnostic reliability enables more frequent screening, earlier identification of wasting, and potentially more timely referral pathways where trained personnel or access to households is limited.

The second theme pertains to acceptance and feasibility. Participant households were able to use the *D2A* app and self-report their children's nutrition status after only two training sessions. Survey completion rates were similar to those in the control group, where CHVs entered the survey data. When households used the app, CHV support significantly increased survey completion rates by 15%, compared to households without CHVs. Dropout spikes were most likely a function of latent conflicts between study participants and other members of the pastoralist community. Pastoralist households that did not participate in the survey data collection were critical of the study and smartphone technologies in general, scrutinizing continued participation of households that signed up for the study in the first place.

And while the association between smartphone use and development outcomes, such as improved gender equality and nutrition (65) or agricultural outcomes (66, 67) is well-documented, qualitative interviews and focus group discussions with participants indicate that the flexibility and agency afforded by self-collection was an important factor in generating sustained participation in the study.

A third theme concerns cost. Relative to face-to-face NDMA surveys, *D2A*-based reporting was substantially less costly per household. Beyond immediate savings, this difference has implications for sustainability: lower per-household expenditures can support higher-frequency data collection and longer-term surveillance. The *D2A* pilot demonstrates the potential for real time information transfer and fast data synchronization, which can effectively be used for early warning, improved targeting, and aid allocation. Given that the same households are interviewed repeatedly, *D2A* data may provide insights regarding the effectiveness of nutrition intervention over time.

### 5.1 Limitations and future work

Whereas our findings provide support for the scalability of autonomous nutrition data collection via smartphone apps, we note three limitations with regard to this proof-of-concept. First, measuring the incidence and intensity of child acute malnutrition has conceptual complexities and practical challenges (68, 69). Self-assessments of child anthropometric information, specifically Family MUAC, are prone to bias (70), measurement error, and can be stressful for children. As the accuracy of collected nutrition data is essential, AI-based technology to accurately measure and diagnose children for their nutrition status (71–73) serves as a plausible alternative to physical scales and measuring boards. In particular, large numbers of high quality images of Family MUAC and hand-measured anthropometric data could be leveraged for the training and validation of machine-learning algorithms, for instance neural networks, that could in turn be used to further simplify the nutrition assessment for participants because children would not even have to be touched. AI-enhanced nutrition assessments could furthermore speed up the review process, taking around 30 seconds per picture.

Second, there are challenges related to certain survey items, in particular a child's age. Future studies could use the child's birth date rather than their age in months to obtain more accurate information. And while our study only sampled children living in households, we rapidly realized that the total number of children is higher due to the fact that most households share child care tasks with their neighbors. It may therefore be worthwhile to include a distinction between “own” children and “children from neighbors and relatives” that do not permanently reside in a household.

A third limitation pertains to issues of scale. While we deem the approach scalable, both within and across national contexts, research into app-based nutrition monitoring would benefit from a larger sample size and the exploration of other geographical regions to confirm the findings of our pilot study. In particular, we note that participants in this research were relatively experienced with the Family MUAC measurement given the existence of nutrition surveillance by NDMA in West Pokot. Although network coverage did not constitute a major concern in this pilot study, smartphone ownership was extremely low, requiring project-provided devices. To scale up an initiative akin to *D2A*, access to affordable smartphones will be essential to reduce digital divide barriers.

We identify two areas of particular interest for future work. First, a closer look at the peculiarities of pastoralist livelihoods and their implications for regular nutrition screenings and data collection. And second, the integration of digital health recommendations and clear referral pathways after each completed “positive” nutrition scan to encourage timely treatment for wasted children. Studies have consistently shown that growth monitoring programs have minimal to no effect on reducing child malnutrition and only lead to improvements when combined with referral to appropriate treatment and behavior change interventions (74, 75).

### 5.2 Integration with existing health information systems

To move beyond a proof-of-concept, long-term institutionalization of app-based nutrition screenings like *D2A* into existing nutrition surveillance and health information systems in Kenya is essential. At the community-level, we recommend leveraging existing support structures to the extent possible. For example, NDMA enumerators who collect representative household level anthropometric data at monthly intervals and CHVs, could (i) introduce the app-based questionnaire during routine household visits or mother support group sessions, (ii) coordinate training on Family MUAC measurement and smartphone use, and (iii) provide additional support to households experiencing difficulty using the app—a pragmatic way to ensure continued participation over the long-term. Alternatively, younger women with sufficient schooling can convey survey instructions to other members of the community, providing meaningful support and guidance beyond initial training sessions. More broadly, we emphasize the importance of dialogue within communities to build a sense of ownership, in an effort to avoid latent conflicts between study participants and non-participants.

At the county and national levels, *D2A* could complement, rather than replace, in-depth face-to-face surveys by filling temporal gaps and providing higher-frequency information on the same households over an extended period of time. *D2A* data, including child nutrition outcomes alongside a few selected household-level risk factors, could in combination with carefully specified and locally validated models, be leveraged for short-term predictions of acute malnutrition prevalence at the level of individual households and communities, and scenario-based forecasts that harness expert knowledge to assess potential outcomes ‘off the equilibrium path.' Tailored analytical outputs could then be made available in NDMA's widely disseminated, monthly Early Warning Bulletins as part of a novel, forward looking section in an existing reporting schedule. A second option for integration is the District Health Information Software 2 (DHIS2) platform, which has been in place since 2011 (76, 77). Caregiver-led nutrition monitoring can meaningfully complement CHV-led activities, and vice versa, thereby fostering better child health outcomes for households and increased local ownership and trust in DHIS2 data for planning and decision-making (78).

## Data Availability

The datasets presented in this study are available on request from the corresponding author.

## Footnotes

1 In West Pokot, phone ownership was extremely low whereas network coverage was high.
