# Supplementary material for: D2A: a community-led smartphone tool for malnutrition screening in Kenya
Source: Front Public Health. 2026 Feb 27;13:1695850. doi: 10.3389/fpubh.2025.1695850 (PMC12983371; doi:10.3389/fpubh.2025.1695850)
Supplement: Supplementary file 1 [file Data_Sheet_1.pdf]

Supplementary Information

D2A: A Community-Led Smartphone Tool for Malnutrition Screening in Kenya

Ravi Bhavnani and Nina Link

Section I: Materials and Methods

Figure S1. Pilot study timeline.

Figure S2. Cheat sheet: Getting started with the *D2A* app.

Table S1. Child-level summary statistics for *D2A* surveys.

Table S2. Household-level summary statistics for *D2A* surveys, by treatment group.

Figure S3. Balance plot illustrating absolute standardized differences in means of baseline conditions and household-level predictors of acute malnutrition before and after 1:1 cardinality matching of Family MUAC observations that were collected via *D2A* and pen-and-paper surveys.

Table S3. Balance table after matching monthly Family MUAC observations that were collected via *D2A* and pen-and-paper surveys, using 1:1 cardinality matching on the ATT.

Table S4. Household-level summary statistics after matching *D2A* and pen-and-paper surveys.

Table S5. Expert interview questionnaire.

Table S6. Summary of FGD topics.

Section II: Accuracy

Figure S4. Overview of missing values in the raw data.

Table S7. Accuracy metrics for Family MUAC categories: *D2A* vs. pen-and-paper benchmark, *D2A* vs. baseline, and pen-and-paper benchmark vs. baseline.

Figure S5. Sensitivity analysis: Confusion matrix for three Family MUAC categories in the *D2A* app and pen-and-paper survey, relative to the West Pokot target.

Table S8. Sensitivity analysis: Paired comparison of sensitivity and specificity for Family MUAC classification accuracy in the *D2A* app and pen-and-paper survey, relative to the West Pokot target.

Section III: Acceptance

Table S9. Differences in completion rates by community.

Table S10. Differences in completion rates by treatment group.

I MATERIALS AND METHODS

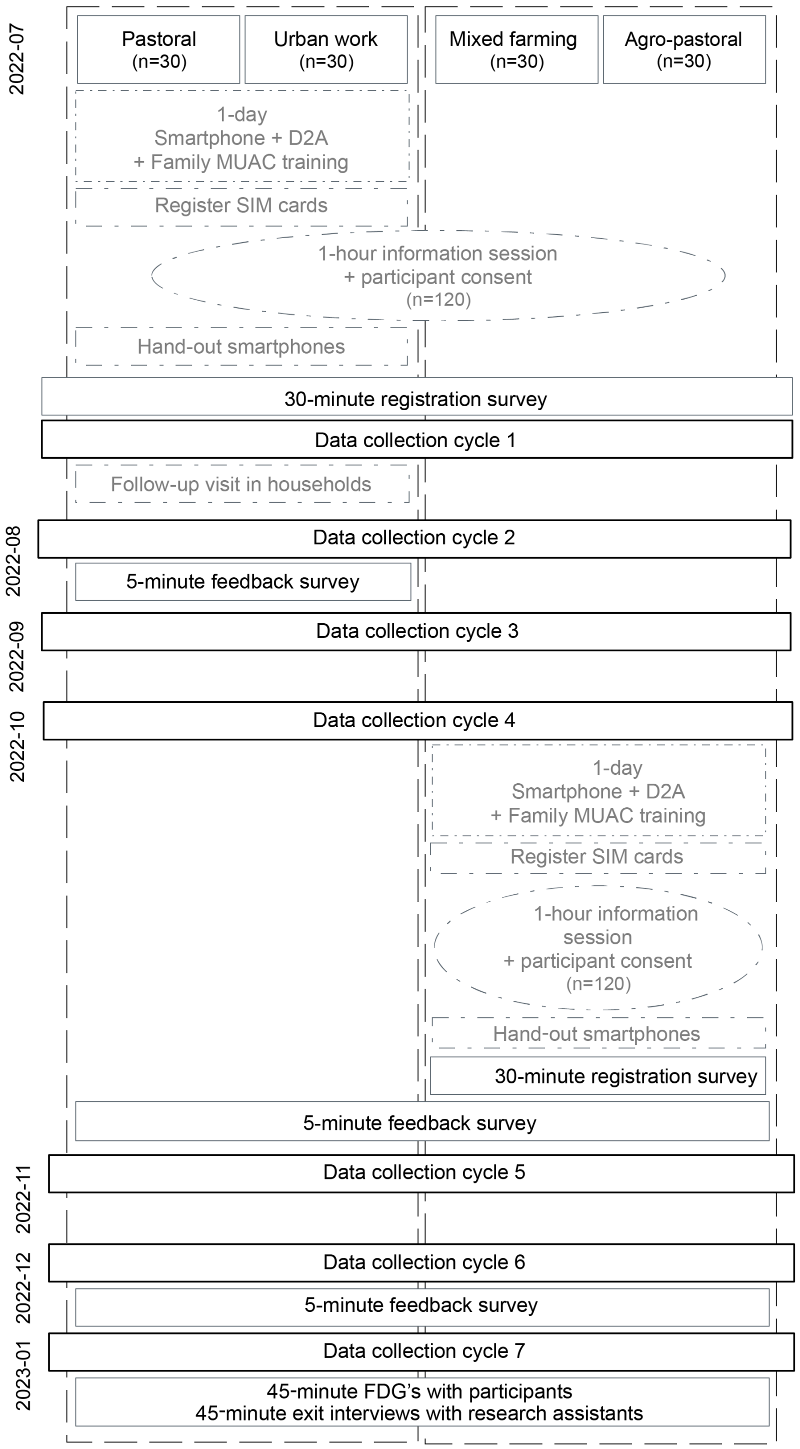

Figure S1. Pilot study timeline.

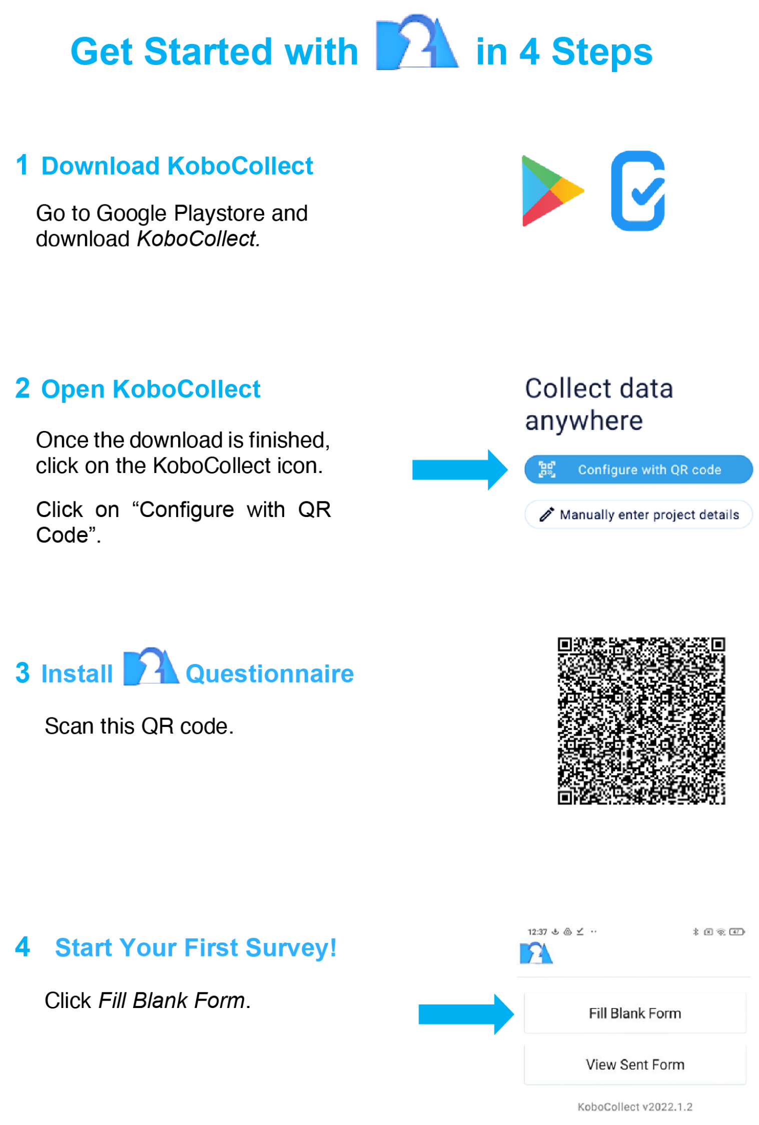

Figure S2. Cheat sheet: Getting started with the D2A app.

Table S1. Child-level summary statistics for *D2A* surveys. Categorical variables are treated as numeric.

	N	Mean	SD	Min	Pctl. 25	Pctl. 75	Max
Grouping variables							
Month							
= Jul 2022	920	0.234	0.423	0	0	0	1
= Aug 2022	920	0.173	0.378	0	0	0	1
= Sep 2022	920	0.14	0.347	0	0	0	1
= Oct 2022	920	0.165	0.372	0	0	0	1
= Nov 2022	920	0.108	0.31	0	0	0	1
= Dec 2022	920	0.0913	0.288	0	0	0	1
= Jan 2023	920	0.0891	0.285	0	0	0	1
Livelihood							
= agro-pastoralists	920	0.221	0.415	0	0	0	1
= pastoralists	920	0.275	0.447	0	0	1	1
= mixed farmers	920	0.254	0.436	0	0	1	1
= street workers	920	0.25	0.433	0	0	0.25	1
Treatment = self-collection							
= false (*pre*)	920	0.316	0.465	0	0	1	1
= true (*post*)	920	0.213	0.41	0	0	0	1
= true	920	0.471	0.499	0	0	1	1
Treatment = community expert							
= present	920	0.496	0.5	0	0	1	1
= absent	920	0.504	0.5	0	0	1	1
Household characteristics							
Education							
= false	920	0.377	0.485	0	0	1	1
= true	920	0.623	0.485	0	0	1	1
Water source							
= safe	920	0.273	0.446	0	0	1	1
= unsafe	920	0.727	0.446	0	0	1	1
Female household head							
= false	920	0.692	0.462	0	0	1	1
= true	920	0.308	0.462	0	0	1	1
Children under 59 months	920	1.58	0.756	1	1	2	3
Income source							
= (casual) labor	920	0.583	0.493	0	0	1	1
= agriculture/farming	920	0.417	0.493	0	0	1	1
Foodstock							
= false	920	0.895	0.307	0	1	1	1
= true	920	0.105	0.307	0	0	0	1
Child nutrition							
Family MUAC category							
= green	920	0.947	0.225	0	1	1	1
= yellow [MAM]	920	0.0446	0.206	0	0	0	1
= red [SAM]	920	0.0087	0.0929	0	0	0	1
Child sex							
= boy	920	0.526	0.5	0	0	1	1
= girl	920	0.474	0.5	0	0	1	1
Child age (months)	920	28	13	6	18	37	59
Child sick							
= false	920	0.943	0.231	0	1	1	1
= true	920	0.0565	0.231	0	0	0	1

Table S2. Household-level summary statistics for *D2A* surveys, by treatment group.

Treatment = self-collection		*false ('pre')*			*true ('post')*			*true*	
	N	Mean	SD	N	Mean	SD	N	Mean	SD
Grouping variables									
Livelihood									
= agro-pastoralists	60	0	0	60	0	0	60	0.5	0.504
= pastoralists	60	0.5	0.504	60	0.5	0.504	60	0	0
= mixed farmers	60	0.5	0.504	60	0.5	0.504	60	0	0
= street workers	60	0	0	60	0	0	60	0.5	0.504
Treatment = community expert									
= present	60	0.5	0.504	60	0.5	0.504	60	0.5	0.504
= absent	60	0.5	0.504	60	0.5	0.504	60	0.5	0.504
Household characteristics									
Max. data collection cycles	60	3.1	1.15	60	2.42	0.809	60	3.98	1.97
Dropout									
= false	60	0.75	0.437	60	0.65	0.481	60	0.3	0.462
= true	60	0.25	0.437	60	0.35	0.481	60	0.7	0.462
Education									
= false	60	0.333	0.475	60	0.517	0.504	60	0.417	0.497
= true	60	0.667	0.475	60	0.483	0.504	60	0.583	0.497
Water source									
= unsafe	60	0.6	0.494	60	0.45	0.502	60	0.95	0.22
= safe	60	0.4	0.494	60	0.55	0.502	60	0.05	0.22
Female household head									
= false	60	0.533	0.503	60	0.567	0.5	60	0.9	0.303
= true	60	0.467	0.503	60	0.433	0.5	60	0.1	0.303
Children under 59 months	60	1.28	0.524	60	1.22	0.415	60	1.38	0.613
Income source									
= (casual) labor	60	0.95	0.22	60	0.85	0.36	60	0.267	0.446
= agriculture/farming	60	0.05	0.22	60	0.15	0.36	60	0.733	0.446
Foodstock									
= false	60	0.917	0.279	60	0.983	0.129	60	0.867	0.343
= true	60	0.0833	0.279	60	0.0167	0.129	60	0.133	0.343

Table S3. Balance table after matching monthly Family MUAC observations that were collected via *D2A* and pen-and-paper surveys, using 1:1 cardinality matching on the ATT.

	Means.Treated	Means.Control	Std.Mean.Diff.	eCDF.Mean	eCDF.Max
Livelihood					
= agro-pastoralists	0.46	0.46	0.01	0.01	0.01
= pastoralists	0.54	0.54	-0.01	0.01	0.01
Month					
= Jul 2022	0.15	0.19	-0.11	0.04	0.04
= Aug 2022	0.15	0.19	-0.11	0.04	0.04
= Sep 2022	0.15	0.14	0.05	0.02	0.02
= Oct 2022	0.15	0.17	-0.07	0.02	0.02
= Nov 2022	0.15	0.12	0.08	0.03	0.03
= Dec 2022	0.15	0.10	0.12	0.04	0.04
= Jan 2023	0.10	0.09	0.03	0.01	0.01
Education					
= false	0.64	0.58	0.13	0.06	0.06
= true	0.36	0.42	-0.13	0.06	0.06
Water source					
= safe	0.42	0.50	-0.14	0.07	0.07
= unsafe	0.58	0.50	0.14	0.07	0.07
Female household head					
= false	0.88	0.47	1.30	0.41	0.41
= true	0.12	0.53	-1.30	0.41	0.41

Table S4. Household-level summary statistics after 1:1 cardinality matching of *D2A* and pen-and-paper surveys from different children in the same communities during the same months. Note that the West Pokot baseline group was down-sampled to N=147 observations—that is the sample size per survey type in the matched data, while keeping the Family MUAC distribution. Categorical variables are treated as numeric.

Treatment = survey		*D2A App*			*Pen-and-Paper*			*Baseline (West Pokot)*	
	N	Mean	SD	N	Mean	SD	N	Mean	SD
Grouping variables									
Livelihood									
= agro-pastoralists	147	0.361	0.482	147	1	0	147	0.0204	0.142
= pastoralists	147	0.639	0.482	147	0	0	147	0.98	0.142
Month									
= Jul 2022	147	0	0	147	0	0	147	0	0
= Aug 2022	147	0	0	147	0	0	147	0	0
= Sep 2022	147	0.0136	0.116	147	0	0	147	0	0
= Oct 2022	147	0.0476	0.214	147	0	0	147	0	0
= Nov 2022	147	0.354	0.48	147	0.374	0.486	147	0.333	0.473
= Dec 2022	147	0.306	0.462	147	0.327	0.471	147	0.333	0.473
= Jan 2023	147	0.279	0.45	147	0.299	0.46	147	0.333	0.473
Household characteristics									
Education									
= false	147	0.633	0.484	147	0.381	0.487	147	0.497	0.502
= true	147	0.367	0.484	147	0.619	0.487	147	0.503	0.502
Water source									
= safe	147	0.612	0.489	147	0.0136	0.116	147	0	0
= unsafe	147	0.388	0.489	147	0.986	0.116	147	1	0
Female household head									
= false	147	0.354	0.48	147	0.878	0.329	147	0.932	0.253
= true	147	0.646	0.48	147	0.122	0.329	147	0.068	0.253
Child nutrition									
Family MUAC category = 'not green'	147	0.0408	0.199	147	0.15	0.358	147	0.0408	0.199
Family MUAC category = yellow [MAM]	147	0.034	0.182	147	0.129	0.337	147	0.034	0.182
Family MUAC category = red [SAM]	147	0.0068	0.0825	147	0.0204	0.142	147	0.0068	0.0825

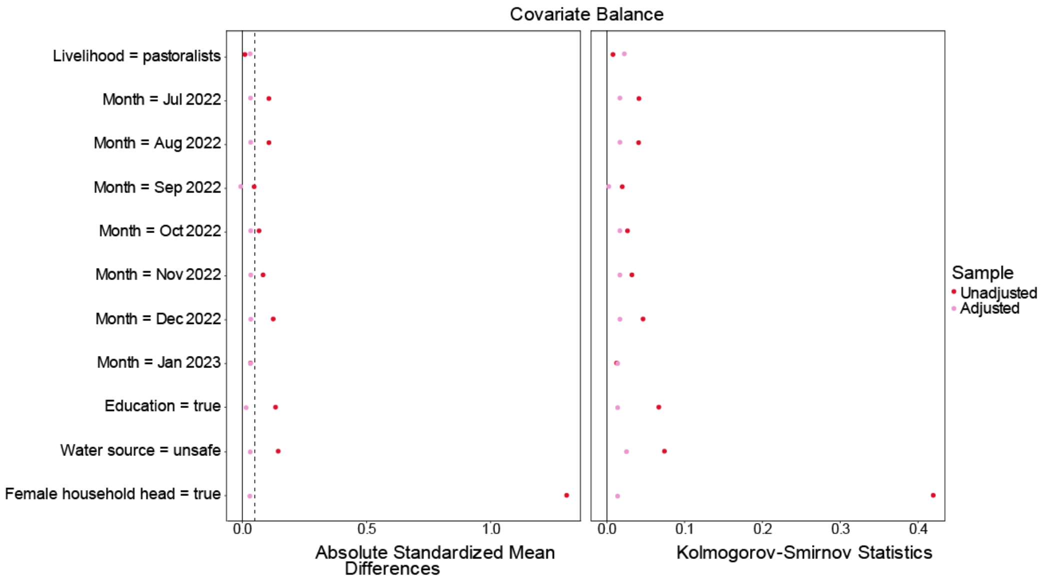

Figure S3. Balance plot illustrating absolute standardized differences in means of baseline conditions and household-level predictors of acute malnutrition before and after 1:1 cardinality matching of *D2A* and pen-and-paper surveys.

Table S5. Expert interview questionnaire.

Question
Is the app-based questionnaire including emojis understandable? If, no: Why?
How could the app interface be improved?
What are in your mind the top 3 challenges with the project?
What are the top three strong points?
Which populations should we target?
For which groups of people would the end product be useful?
How would these groups use it?
Do you know of other initiatives that are comparable to this one?
Who else should we talk to?
Is there anything else you would like to tell us?

Table S6. Summary of FGD topics.

Question
Did you like the study? Why or why not?
What is most useful about D2A?
What is most least useful about D2A?
Would you use D2A after the end of this study? Why?
Which nutrition screening do you prefer? Paper-based or app-based?
Have you used a smartphone before?
Does someone you know own a smartphone?
Who in your household uses D2A?
Do you use it alone or with others?
What has changed since you use D2A?

II ACCURACY

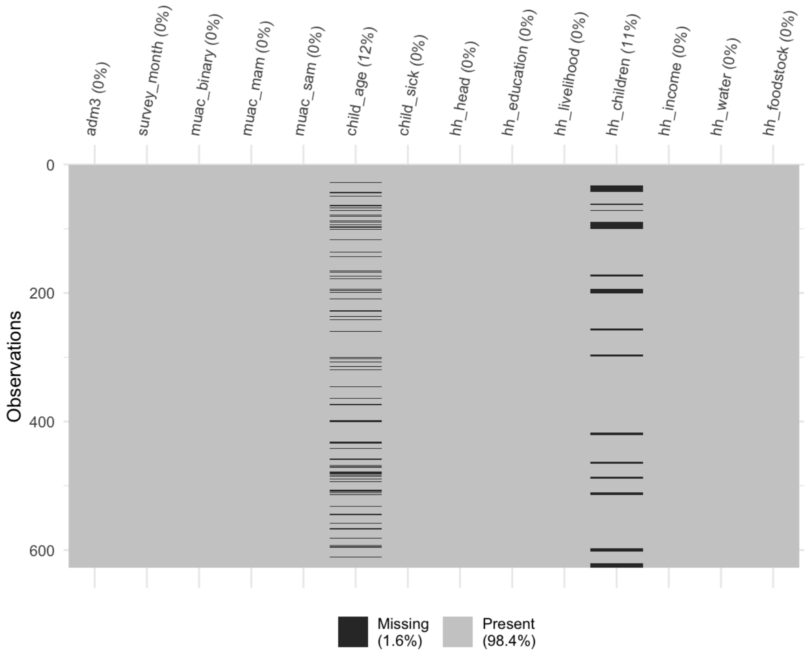

Figure S4. Overview of missing values in the raw data.

Table S7. Accuracy metrics for Family MUAC categories: *D2A* vs. pen-and-paper benchmark, *D2A* vs. baseline, and pen-and-paper benchmark vs. baseline.

Family MUAC Cat.	Prediction	Target	Acc. Measure	Est.	SE	$95\%\text{CI}_{Lower}$	$95\%\text{CI}_{Upper}$
not green	D2A App	Baseline	Sensitivity	0.00	0.00	0.00	0.00
not green	Pen-and-paper	Baseline	Sensitivity	0.17	0.15	0.00	0.47
not green	D2A App	Baseline	Specificity	0.96	0.02	0.92	0.99
not green	Pen-and-paper	Baseline	Specificity	0.85	0.03	0.79	0.91
not green	D2A App	Baseline	Pos. Pred. Value	0.00	0.00	0.00	0.00
not green	Pen-and-paper	Baseline	Pos. Pred. Value	0.04	0.04	0.00	0.13
not green	D2A App	Baseline	Neg. Pred. Value	0.96	0.02	0.92	0.99
not green	Pen-and-paper	Baseline	Neg. Pred. Value	0.96	0.02	0.93	0.99
yellow [MAM]	D2A App	Baseline	Sensitivity	0.00	0.00	0.00	0.00
yellow [MAM]	Pen-and-paper	Baseline	Sensitivity	0.20	0.18	0.00	0.55
yellow [MAM]	D2A App	Baseline	Specificity	0.96	0.01	0.93	0.99
yellow [MAM]	Pen-and-paper	Baseline	Specificity	0.87	0.03	0.82	0.93
yellow [MAM]	D2A App	Baseline	Pos. Pred. Value	0.00	0.00	0.00	0.00
yellow [MAM]	Pen-and-paper	Baseline	Pos. Pred. Value	0.05	0.05	0.00	0.15
yellow [MAM]	D2A App	Baseline	Neg. Pred. Value	0.96	0.01	0.93	0.99
yellow [MAM]	Pen-and-paper	Baseline	Neg. Pred. Value	0.97	0.01	0.94	1.00
red [SAM]	D2A App	Baseline	Sensitivity	0.00	0.00	0.00	0.00
red [SAM]	Pen-and-paper	Baseline	Sensitivity	0.00	0.00	0.00	0.00
red [SAM]	D2A App	Baseline	Specificity	0.99	0.01	0.98	1.00
red [SAM]	Pen-and-paper	Baseline	Specificity	0.98	0.01	0.96	1.00
red [SAM]	D2A App	Baseline	Pos. Pred. Value	0.00	0.00	0.00	0.00
red [SAM]	Pen-and-paper	Baseline	Pos. Pred. Value	0.00	0.00	0.00	0.00
red [SAM]	D2A App	Baseline	Neg. Pred. Value	0.99	0.01	0.98	1.00
red [SAM]	Pen-and-paper	Baseline	Neg. Pred. Value	0.99	0.01	0.98	1.00

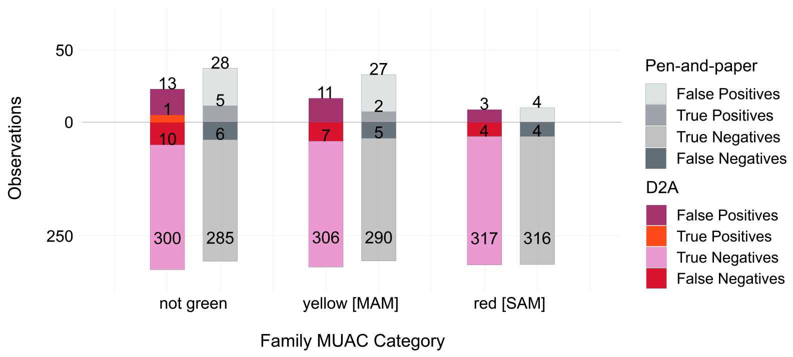

Figure S5. Sensitivity analysis: Confusion matrix for three Family MUAC categories in the *D2A* app and pen-and-paper survey, relative to the West Pokot target. Up-sampling to 610 observations was used for the *D2A* and pen-and-paper samples, to meet the number of observations in the West Pokot target.

Table S8. Sensitivity analysis: Paired comparison of classification accuracy in self-reported Family MUAC measures via the *D2A* app and pen-and-paper surveys, *between* livelihood groups and relative to the West Pokot baseline.

	Family MUAC Cat.	D2A App	Pen-and-paper	Diff.	Diff. SE	95%CI_{Lower}	95%CI_{Upper}
Sensitivity	'not green'	0.182	0.091	-0.091	0.087	-0.261	0.079
	yellow [*MAM*]	0.143	0.143	0	0	0	0
	red [*SAM*]	0	0	0	0	0	0
Specificity	'not green'	0.965	0.901	-0.064	0.014	-0.091	-0.037
	yellow [*MAM*]	0.972	0.918	-0.054	0.013	-0.079	-0.029
	red [*SAM*]	0.991	0.984	-0.006	0.004	-0.015	0.002

III ACCEPTANCE

Table S9. Differences in completion rates by community.

	Group 1	Group 2	N(1)	N(2)	*p*	*p* signif.	*p* adj.	*p* adj. signif.
1	Pastoralists	Mixed farmers	153	191	0.01	**	0.03	*
2	Pastoralists	Street workers	153	124	0.21	ns	1.00	ns
3	Mixed farmers	Street workers	191	124	0.00	****	0.00	***
4	Pastoralists	Agro-pastoralists	153	220	0.70	ns	1.00	ns
5	Mixed farmers	Agro-pastoralists	191	220	0.01	**	0.05	*
6	Street workers	Agro-pastoralists	124	220	0.09	ns	0.53	ns

Table S10. Differences in completion rates by treatment group.

Group 1	Group 2	df	*p*	Est. Diff.	95%CI_{Lower}	95%CI_{Upper}
Self-collection= *false ['pre']*	Self-collection= *true ['post']*	59	5.299	0.228	0.123	0.332
Self-collection= *false ['pre']*	Self-collection= *true*	59	0.744	-0.022	-0.158	0.113
